# Preliminary Data on the Antiviral Activity of *Helleborus bocconei* subsp. *intermedius* Root Extracts Against Animal Herpesviruses

**DOI:** 10.3390/microorganisms13040891

**Published:** 2025-04-12

**Authors:** Paola Galluzzo, Santina Di Bella, Sergio Migliore, Maria Valeria Raimondi, Roberta Bivacqua, Gigliola Borgonovo, Salvatore Princiotto, Antonella Girgenti, Laura Palumbo, Salvatore Dara, Annalisa Guercio, Rosa Alduina, Guido Ruggero Loria, Vincenza Cannella

**Affiliations:** 1 Istituto Zooprofilattico Sperimentale della Sicilia “A. Mirri”, Via G. Marinuzzi 3, 90129 Palermo, Italy; paola.galluzzo@izssicilia.it (P.G.); sergio.migliore@izssicilia.it (S.M.); salvatore.dara@izssicilia.it (S.D.); annalisa.guercio@izssicilia.it (A.G.); guidoruggero.loria@izssicilia.it (G.R.L.); vincenza.cannella@izssicilia.it (V.C.); 2 Dipartimento Scienze e Tecnologie Biologiche, Chimiche e Farmaceutiche, Viale delle Scienze, University of Palermo, 90133 Palermo, Italy; mariavaleria.raimondi@unipa.it (M.V.R.); roberta.bivacqua@unipa.it (R.B.); valeria.alduina@unipa.it (R.A.); 3 Department of Food, Environmental and Nutritional Sciences, Section of Chemical and Biomolecular Sciences, University of Milano, 20133 Milano, Italy; gigliola.borgonovo@unimi.it (G.B.); salvatore.princiotto@unimi.it (S.P.); 4 Institute for Biomedical Research and Innovation, National Research Council of Italy, Via U. La Malfa, 153, 90146 Palermo, Italy; antonella.girgenti@irib.cnr.it (A.G.); laura.palumbo@cnr.it (L.P.)

**Keywords:** *Helleborus bocconei*, bovine alphaherpesvirus-1 (BoAHV1), caprine alphaherpesvirus-1 (CpAHV1), equine alphaherpesvirus-1 (EqAHV1)

## Abstract

*Orthoherpesviridae* is a large family of enveloped DNA virus. Among the most significant animal-infecting viruses are bovine alphaherpesvirus 1 (BoAHV1), caprine alphaherpesvirus 1 (CpAHV1) and equid alphaherpesvirus 1 (EqAHV1). Research into new methods to combat herpesvirus infections is ongoing. The aim of this study was to evaluate the antiviral activity of three extracts of the *Helleborus bocconei* roots against BoAHV1, CpAHV1 and EqAHV1. The roots were air-dried, extracted with methanol (MeOH) and then partitioned between *n*-butanol (*n*-BuOH) and water. All three extracts were tested for cytotoxicity on MDBK and RK-13 cells, and for antiviral activity. Two non-cytotoxic concentrations were assessed for their anti-BoAHV1, anti-CpAHV1 and anti-EqAHV1effects. Cells were incubated with the extracts for 72 h under three experimental conditions: pretreatment before viral infection, treatment post virus infection and simultaneous viral infection and treatment with extracts. The *n*-BuOH extract (BE) at 0.62 µg/mL inhibited the cytopathic effects of all three viruses in the simultaneous assay. Additionally, no cytopathic effect was observed in MDBK cells infected with CpAHV1and treated with 0.31 µg/mL BE post virus infection. Therefore, the BE contains molecules or groups of molecules potentially useful for developing an alternative therapy against herpesvirus (HV) infection.

## 1. Introduction

*Orthoherpesviridae* is a large family of enveloped DNA virus spread worldwide and causing health problems in humans and animals [1]. The family is divided into three subfamilies, named *Alpha*-, *Beta*- and *Gammaherpesvirinae* [2]. In particular, alphaherpesviruses have a variable host range and are characterized by rapid, lytic growth cycles and primarily establish latency in neurons of sensory ganglia [3]. Infection caused by alphaherpesviruses primarily occurs via the respiratory tract through aerosolized virus particles, whereas the genital tract involvement typically follows viremia resulting from the initial respiratory infection [3].

Among the most clinically relevant herpesviruses infecting animals are bovine alphaherpesvirus 1 (BoAHV1), caprine alphaherpesvirus 1 (CpAHV1) and equid alphaherpesvirus 1 (EqAHV1). These viruses cause high economic losses [4,5,6] mainly due to abortions of infected animals and neonatal diseases, as well as expenses for the treatment of animals with respiratory and neurological clinical signs or the death of animals [7]. 

All three viruses are associated with respiratory and reproductive symptoms (reproductive failure, abortions and stillbirth) and tend to induce latent infections in the trigeminal and sacral ganglia [1,7,8,9,10]. Furthermore, BoAHV1 and EqAHV1 have been implicated in neurological disorders including encephalitis [1,11,12,13].

Current therapeutic strategies for managing herpesvirus infections in animals remain limited and often fail to fully prevent or control viral spread [14,15]. Vaccination is the primary preventive measure, yet it has limitations. Although available vaccines effectively reduce the severity of clinical symptoms and viral shedding, they do not provide complete immunity against infection or reactivation of latent viruses [16]. This is particularly problematic in animal populations, where latent viruses can persist in sensory ganglia and reactivate under stress conditions (such as climate changes, transportation, or corticosteroid treatments). As a result, animals may remain asymptomatically infected for extended periods, only to experience viral reactivation and subsequent outbreaks [17].

Regarding antiviral treatment, there is a lack of effective options for herpesvirus infections in animals. Although nucleoside analogs, such as acyclovir, show some efficacy in humans, their use in veterinary medicine is limited, often due to restricted approval across species, variable efficacy in preventing reactivation, and high costs. Furthermore, long-term use of such antiviral agents in animals is not always feasible or effective in managing recurrent infections [18].

Current management strategies predominantly focus on symptomatic treatment, such as addressing respiratory or neurological signs, often with antibiotics to prevent secondary infections or antiinflammatory drugs to mitigate symptoms. However, these approaches do not target the virus directly and are insufficient in preventing viral reactivation or transmission.

The limitations of existing vaccines and antiviral therapies contribute to substantial economic losses in the livestock industry due to reproductive failures, respiratory diseases, and neurological complications, in addition to rising veterinary costs.

Given these constraints, there is an urgent need for alternative therapeutic approaches [19]. Although the primary focus has been on synthetic products, the study of natural compounds with antiviral action for different DNA and RNA viruses is increasing [20]. Plant-derived antiviral agents, particularly those from natural sources, offer a promising pathway for the development of adjunctive or alternative treatments [21]. Natural agents could complement existing vaccines or provide independent therapeutic options to reduce viral load and enhance overall disease management. The exploration of plant-based antiviral compounds, such as those from *Helleborus* species, offers a promising direction for future research and may help address the ongoing challenges associated with herpesvirus infections in veterinary medicine.

*Helleborus* is a genus of herbaceous perennials belonging to the family *Ranunculaceae*, comprises around 20 species and includes perennial herbs native to Europe and Asia [22].

Roots, rhizomes and leaves are still used in traditional medicine to treat humans and animals. The rhizomes contain starch granules and oleosomes accumulate the largest amount of metabolites that can have a broad spectrum of pharmacological and therapeutic effects [23]: these plants are rich in structurally diverse active compounds, such as cardiac glycosides, steroidal saponins, ecdysones, and protoanemonin [23,24,25]. *Helleborus* species have demonstrated pharmacological effects including antirheumatic [26], antiinflammatory [27,28,29], anticancer [30,31], antidiabetic, antibacterial [32], and antioxidant properties [33,34] but their ability as antiviral has not been yet investigated.

In the context of Italian ethnoveterinary medicine, *Helleborus foetidus* and *Helleborus viridis* have primarily been utilized for the treatment of various pathological conditions affecting swine, bovines, ovines, mules, and donkeys [35,36]. In particular, *Helleborus bocconei* subsp. *intermedius* has been traditionally applied for the management of respiratory diseases such as pneumonia and bronchitis in cattle [37]. The therapeutic practice typically involves making small dermal incisions into which fragments of the dried root are inserted. It is reported that, upon subsequent removal of both the root material and associated purulent matter, clinical recovery is generally observed within 5 to 6 days [32]. In light of these practices, this study aimed to investigate the biological activity of methanol (MeOH), *n*-butanol (*n*-BuOH), and aqueous extracts of *H. bocconei* roots, collected in Sicily (Southern Italy). The evaluation focused on their cytotoxicity in MDBK and RK-13 cell lines, as well as their antiviral efficacy against animal herpesviruses, specifically BoAHV1, CpAHV1 and EqAHV1. The findings provide a valuable contribution to understanding the pharmacological potential of *H. bocconei*, support its traditional use, and suggest further investigation of its bioactive compounds for the development of novel veterinary therapeutics.

## 2. Materials and Methods

### 2.1. Plant Collection, Extraction and Purification Procedures

*Helleborus bocconei* plants were harvested in the area around Alcara Li Fusi (about 1300 m above sea level), Messina (Italy). Roots were air dried at room temperature for a period of 30 days afterwards were washed, powdered and stored at room temperature until the extraction.

An amount of 35 g of *H. bocconei* roots were sequentially extracted sequentially with two 350 mL aliquots of MeOH each, by shaking for 3 h at room temperature in the absence of light. After the first extraction, the decanted suspension was filtered, and the solid part was extracted again. The two extracts were combined, and the organic solvent was removed in the rotavapor maintaining the water bath temperature at 30 °C. Finally, nitrogen was blown and 10 g of MeOH crude extract (ME) was obtained as a clear brown oil. Finally, 1.0 g of ME was partitioned between *n*-BuOH and water (3:1 *v*/*v*) for three times to obtain, after evaporation of the solvents, 0.55 g of aqueous extract (AE) and 0.45 g of *n*-BuOH extract (BE) [38].

**Figure 1:**
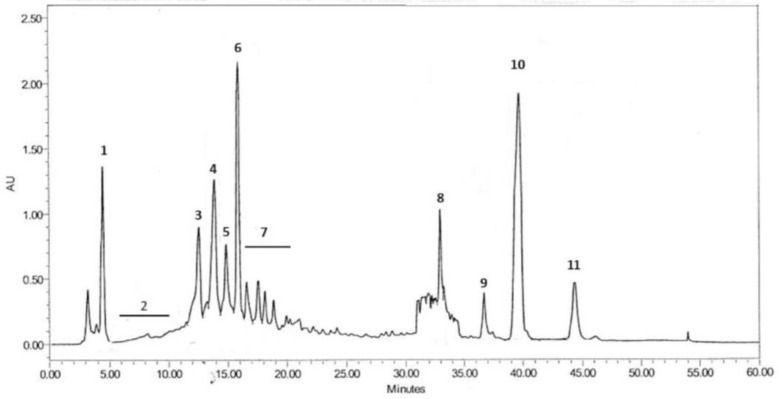
HPLC fingerprint of BE (the number of fractions is indicated in bold type).

Aqueous extract analyzed by ^1^H NMR resulted to contain mainly carbohydrates, identifiable by the presence of diagnostic signals in the central region of the spectrum between 3.2 and 5.4 ppm. BE was purified by preparative HPLC. A 10 mg/mL stock solution of BE was prepared by dissolving the extract in a mixture of acetonitrile (ACN) and water 3:7. The solution was filtered using a nylon syringe filter of 0.22 μm size and separated by preparative HPLC. Preparative HPLC was performed with a C-18 Ascentis column, Supelco (21.2 i.d._250 mm, 5 µm), fitted to a 1525 Extended Flow Binary HPLC pump and a Waters 2489 UV–Vis detector (both from Waters, Milan, Italy). The mobile phase consisted of solvent A (water) and solvent B (ACN); eluent mixture composition was changed from 10% to 60% of solvent B over 15 min, kept at 60% until 25 min and raised to 100% at 30 min. The flow rate was 15 mL/min, the volume of injection 1 mL and the detector was set at 210 and 280 nm. The fractions corresponding to the major peaks, 1–11 (Figure 1), were collected and the solvent was removed in vacuum.

NMR spectra were performed on a Bruker AV600 spectrometer, equipped with a 5 mm triple-resonance probe with *z*-axis gradients and variable temperature control unit. The analyses were performed at 25 °C. Spectra were referenced on external TMS, set at 0 ppm. Spectra were transformed and analyzed with the aid of TOPSPIN (v.4.0.1, Bruker Spectrospin, Germany). Structures of molecules were identified by 1D and 2D NMR.

### 2.2. Cell Lines

Madin Darby bovine kidney (MDBK; ATCC CCL-22) and rabbit kidney (RK-13; ATCC CCL-37) cell lines, were maintained in Eagle’s minimal essential medium (E-MEM) supplemented with an antibiotic and antimycotic solution (100 U/mL penicillin G sodium salt, 0.1 mg/mL streptomycin sulfate, 0.25 μg/mL amphotericin B; EuroClone^®^, Milan, Italy), and 10% fetal bovine serum (FBS, EuroClone^®^, Milan, Italy). A total of 100 μL of suspension containing 1 × 10^3^ cells were seeded into each well of a 96-well culture plate for both cytotoxicity and antiviral assays. All plates were incubated at 37 °C in a 5% CO_2_ atmosphere.

### 2.3. Viruses

Virus stocks of BoAHV1 and CpAHV1 were propagated in MDBK cell cultures. EqAHV1 was propagated in RK-13 cell cultures. MDBK and RK-13 cells were also used for the measurement of viral infectivity by a dilution method using a 96-well microtiter plate. The infection titers were expressed as 50% tissue culture infectious doses (TCID_50_) calculated by Reed and Muench method, as previously described [39]. The BoAHV1 strain with a mean titer of 10^6.79^ TCID_50_/mL, the EqAHV1 strain with a mean titer of 10^5.36^ TCID_50_/mL and the CpAHV1 strain with a mean titer of 10^3.67^ TCID_50_/mL were used.

### 2.4. Cytotoxicity Assays

Before assessing the potential antiviral activity of the three *H. bocconei* extracts, their cytotoxic effects on MDBK cells (used for the cultivation of BoAHV1 and CpAHV11) and RK-13 cells (used for the cultivation of EqAHV1) were examined. A total of 100 μL of a cell suspension containing 1 × 10^3^ cells was seeded into each well of a 96-well plate and incubated at 37 °C in a 5% CO_2_ atmosphere. After 24 h, 200 μL of each of the three *H. bocconei* extracts (ME, BE and AE), prepared in E-MEM with 1% DMSO, at concentrations ranging from 0.31 μg/mL to 25 μg/mL, were analyzed in triplicate. Phenol (0.5%) was used as a positive control for cytotoxicity in all experiments. Cell monolayers cultured in E-MEM were used as a cell control.

Cytotoxicity was determined by the MTT (3-(4,5-dimethylthiazole-2-yl)-2,5-diphenyl tetrazolium bromide) colorimetric method at 24, 48 and 72 h. The Cell Titer 96 Aqueous One Solution Reagent (Promega Italia s.r.l., Milan, Italy) (20 µL) was added, and the plate was maintained for 4 h at 37 °C in a humidified 5% CO_2_ atmosphere. Plates were read on a microplate spectrophotometer reader (Sunrise™, Tecan, Cernusco sul Naviglio (MI), Italy), equipped with reader control and data analysis software (Magellan™ v7.2 software, Tecan, Cernusco sul Naviglio (MI), Italy) at a wavelength of 492 nm. The percentage of cell survival was calculated. The experiment was conducted in triplicate to determine both the extract concentration that induced 50% cell death (CC50) and the maximum non-toxic concentration (MNTC), which is the highest extract concentration that does not induce cell death.

Furthermore, cell growth, morphology and viability were assessed under a light microscope at X100 magnification. Cytotoxicity was detected when degenerated cells became round and floated in the medium with more prominent nuclei.

### 2.5. Antiviral Assays

Two non-cytotoxic concentrations below the MNTC of each plant extract were tested, in triplicate, to evaluate their anti-BoAHV1, anti-CpAHV1 and anti-EqAHV1 activity.

In order to assess the antiviral activity, cells were incubated with the extracts for 72 h under three experimental conditions: i. pretreatment of cells with the extracts prior to viral infection (Method A); ii. treatment with the extracts post viral infection (Method B); and iii. simultaneous viral infection and treatment with the plant extracts (Method C) [40,41]. In all three methods (A, B and C), the following control groups were included: (1) untreated infected cells (virus control), consisting of confluent monolayer cells infected with the virus at 10^2^ TCID_50_ mL^−1^; (2) uninfected cells treated with plant extracts (plant extract controls); and (3) uninfected untreated cells (cell control). After 72 h of incubation, optical density (OD) was measured as described previously. The antiviral activity of the plant extracts, after each of the follow described methods, was calculated using the following formula:

Antiviral activity (%) = 100 × (Abs sample − Abs virus control)/(Abs cell control − Abs virus control)

The absorbance (Abs) of the control virus was determined by adding 200 μL of virus suspension (10^2^ TCID_50_) without plant extracts. The absorbance of plant extract controls was measured by adding a total volume of 200 μL, consisting of culture medium and *H. bocconei* extract, to reach the respective MNTC for each extract.

#### 2.5.1. Cell Culture Pretreatment with *H. bocconei* Extracts (Method A)

Pretreatment of cell cultures was performed by exposing the cell monolayers to different concentrations of the *H. bocconei* extracts in maintenance medium (200 μL) for 2 h at 37 °C before virus infection. After treatment, medium was removed and the cell monolayers were washed thoroughly with Hank’s balanced salt solution (HBSS, Sigma–Aldrich^®^, Milan, Italy), infected with 200 μL of BoAHV1, CpAHV1 or EqAHV1 suspension at 10^2^ TCID_50_ mL^−1^ of virus, and after 72 h of incubation at 37 °C in a 5% CO_2_ atmosphere viral cytopathic activity was observed.

#### 2.5.2. Treatment with *H. bocconei* Estracts Post Virus Infection (Method B)

An aliquot of 200 μL of viral suspension at 10^2^ TCID_50_ mL^−1^ in culture medium was adsorbed to MDBK or RK-13 cells for 2 h at 37 °C. After incubation, cells were washed with HBSS, and the medium was replaced with 200 μL of E-MEM containing serial dilutions of the ME, BE and AE of *H. bocconei*. The cells were then incubated for 72 h at 37 °C in a 5% CO_2_ atmosphere.

#### 2.5.3. Simultaneous Viral Infection and Treatment of Cell Cultures with *H. bocconei* Extracts (Method C)

In the simultaneous infection and treatment, virus suspensions at a concentration of 10^2^ TCID_50_ mL^−1^ were mixed with varying concentrations of *H. bocconei* extracts at room temperature. The resulting mixtures were then immediately added to cells (200 μL per well) and incubated for 72 h at 37 °C in a 5% CO_2_ atmosphere.

### 2.6. Statistical Analysis

The data are presented as means ± standard deviation (S.D). The T-test was used to compare differences between mean groups. A *p*-value < 0.05 was considered to imply statistical significance. The statistical analysis was performed using GraphPad package (version 8.1).

## 3. Results

### 3.1. Chemical Composition of BE

A total of 11 fractions were isolated by preparative HPLC (Figure 1) and analyzed by NMR. Fractions 1 and 2 contain a complex mixture of unidentified compounds, fraction 3 contains a mixture of ecdysterones (ecdysone and 20-hydroxyecdisone; NMR data are in agreement with those reported by Girault and Lafont) [42]. Fraction 4 contains helleborosides A and B (NMR data are in agreement with those reported by Rosselli et al.) [38]. Fraction 5 mainly contains bufadienolides, whereas fraction 6 shows NMR signals consistent to hellebrigenin according to van Heerden et al. [43]. Fraction 7 contains helleborosides and bufadienolides. Complex mixtures of aliphatic and unsaturated fatty components are present in fractions 8–11.

### 3.2. Cytotoxicity of H. bocconei Extracts

To examine the effect of *H. bocconei* extracts on the growth and the viability of MDBK and RK-13 cell cultures, the ME, BE and AE were serially diluted (from 25 μg/mL to 0.31 μg/mL) and added to cell culture medium.

Cell viability of MDBK cells was reduced at concentrations higher than 1.25 μg/mL for the ME and AE and 0.62 μg/mL for BE. Regarding RK-13 cells, their viability was reduced at concentrations higher than 2.5 μg/mL for ME and AEs and 0.62 μg/mL for BE (Table 1).

The CC_50_ values determined by the colorimetric assay in MDBK and RK-13 cells were reported in Table 2.

### 3.3. Antiviral Assays

Two non-cytotoxic concentrations of each extract (0.31 and 0.62 μg/mL for the BE, 0.62 and 1.25 μg/mL for both the ME and AE) were tested to assess their activities anti-BoAHV1, anti-CpAHV1 and anti-EqAHV1. Cells were incubated with the extracts for 72 h, following three experimental conditions: cell culture pretreatment with *H. bocconei* extracts, treatment with extracts post virus infection, and simultaneous viral infection and treatment with extracts.

#### 3.3.1. Antiviral Assays Against BoAHV1

In the antiviral assays against BoAHV1, pretreatment of the cells with *H. bocconei* extracts, followed by viral infection (Method A), did not induce significant changes in cell viability compared to untreated infected cells, except for 0.31 μg/mL BE, which resulted in a slight increase.

Treatment with *H. bocconei* extracts post virus infection (Method B) led to only modest increases in cell viability, with the most notable effect observed for 1.2 μg/mL ME and 0.62 μg/mL BE.

**Figure 2:**
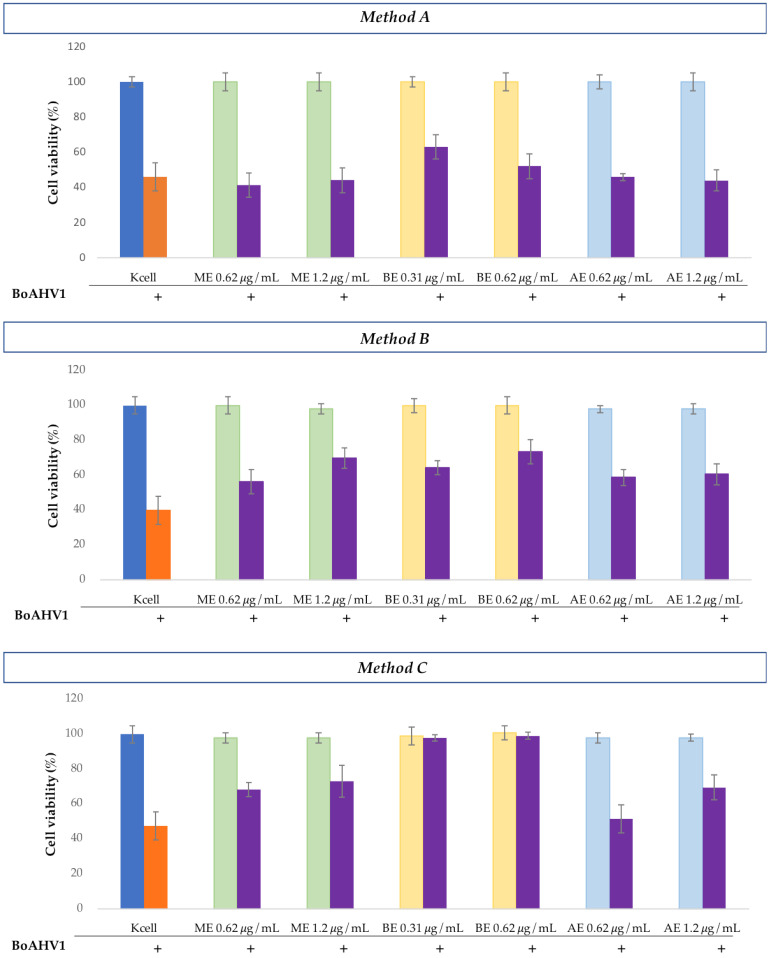
Cell viability (%) of MDBK cells observed in the three experiments: **Method A**: Cell culture pretreatment with *H. bocconei* extracts; **Method B**: Treatment with *H. bocconei* extracts post virus infection; **Method C**: Simultaneous viral infection and treatment of cell cultures with *H. bocconei* extracts. Blue bar: MDBK not treated; Orange bar: MDBK infected with BoAHV1; Purple bar: cells treated with two different concentrations of each *H. bocconei* extract and infected with BoAHV1; Green bar: control of MDBK cells treated with ME; Yellow bar: control of MDBK cells treated with BE and Light blue: control of MDBK cells treated with AE.

**Figure 3:**
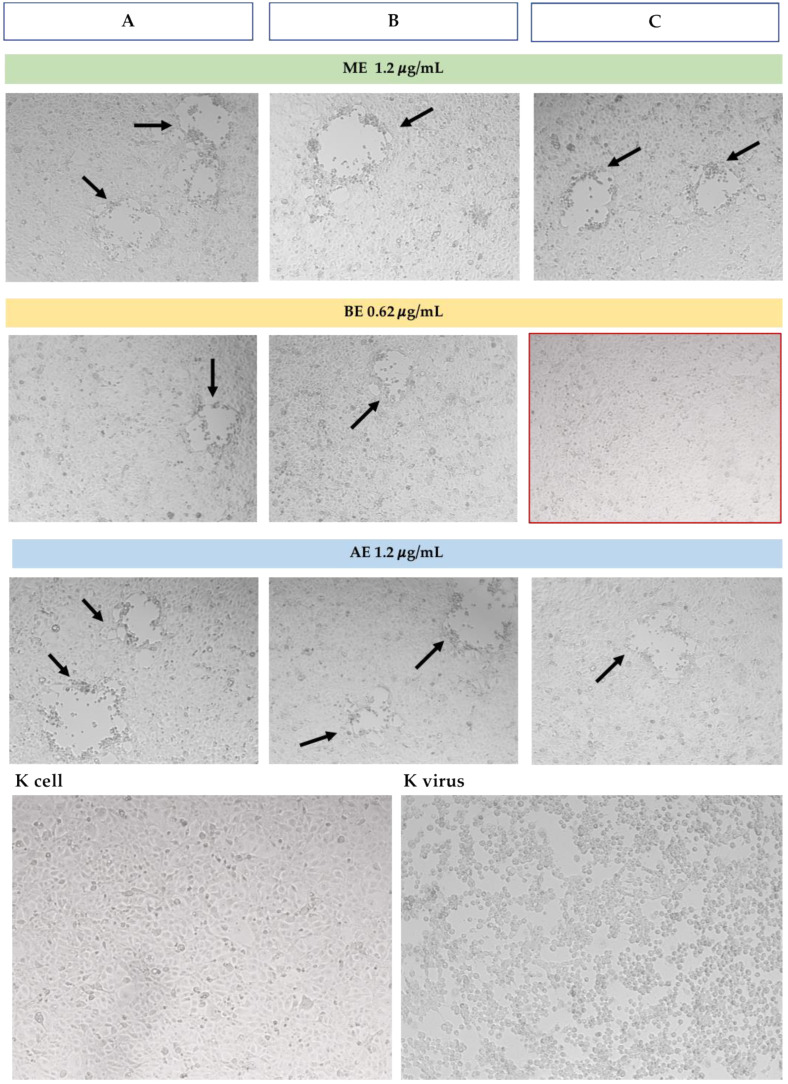
Microscopic observation of MDBK cell morphology (10×) in the three assays with the higher concentration of *H. bocconei* extracts. K cell: cell control (untreated), K virus: virus control (cells with BoAHV1). Black arrows indicate zones with cytopathic effect induced by BoAHV1. The red frame indicates the absence of cytopathic effect. Method (**A**): Cell culture pretreatment with *H. bocconei* extracts; (**B**): Treatment with *H. bocconei* extracts post virus infection; (**C**): Simultaneous viral infection and treatment of cell cultures with *H. bocconei* extracts.

In simultaneous treatment with *H. bocconei* (Method C), 0.31 and 0.62 μg/mL BE improved cell viability: no significant differences were detected between cell viability of virus-infected and virus-uninfected cells. More modest increases were also observed with ME at both tested concentrations and with AE at the highest concentration (Figure 2). Cell morphology analysis was consistent with the increase in MDBK cell viability, as revealed by the MTT assay. Notably, the BE inhibited the cytopathic effect induced by BoAHV1 (Figure 3), preserving the integrity of the cell monolayer.

#### 3.3.2. Antiviral Assays Against CpAHV1

In the assays conducted on MDBK cells infected with CpAHV1, pretreatment with *H. bocconei* extracts (Method A), resulted in a slight increase in cell viability only after treatment with 0.31 μg/mL BE.

In the treatment with *H. bocconei* extracts post virus infection (Method B), MDBK cells treated with both concentrations of BE and AE, as well as those treated with 1.2 μg/mL ME, showed an increase in cell viability compared to infected cells.

**Figure 4:**
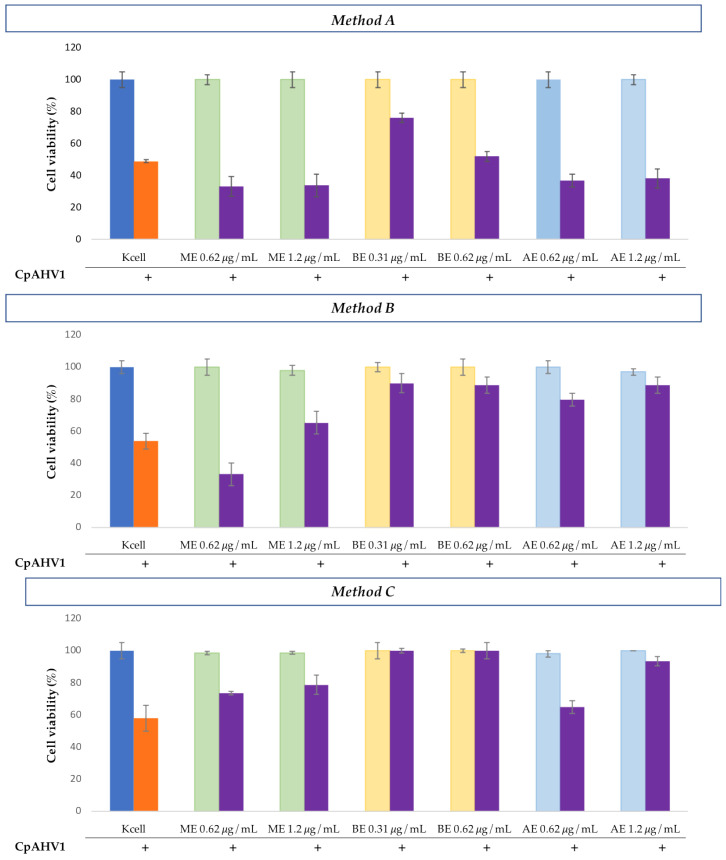
Cell viability (%) of MDBK cells observed in the three experiments: **Method A**: Cell culture pretreatment with *H. bocconei* extracts; **Method B**: Treatment with *H. bocconei* extracts post virus infection; **Method C**: Simultaneous viral infection and treatment of cell cultures with *H. bocconei* extracts. Blue bar: MDBK not treated; Orange bar: MDBK infected with CpAHV1; Purple bar: cells treated with two different concentrations of each *H. bocconei* extract and infected with CpAHV1; Green bar: control of MDBK cells treated with ME; Yellow bar: control of MDBK cells treated with BE and Light blue: control of MDBK cells treated with AE.

Regarding simultaneous infection with CpAHV1 and treatment with *H. bocconei* (Method C), MDBK cells treated with BE showed high cell viability percentages, while only a slight increase was observed with ME and AE treatments (Figure 4).

**Figure 5:**
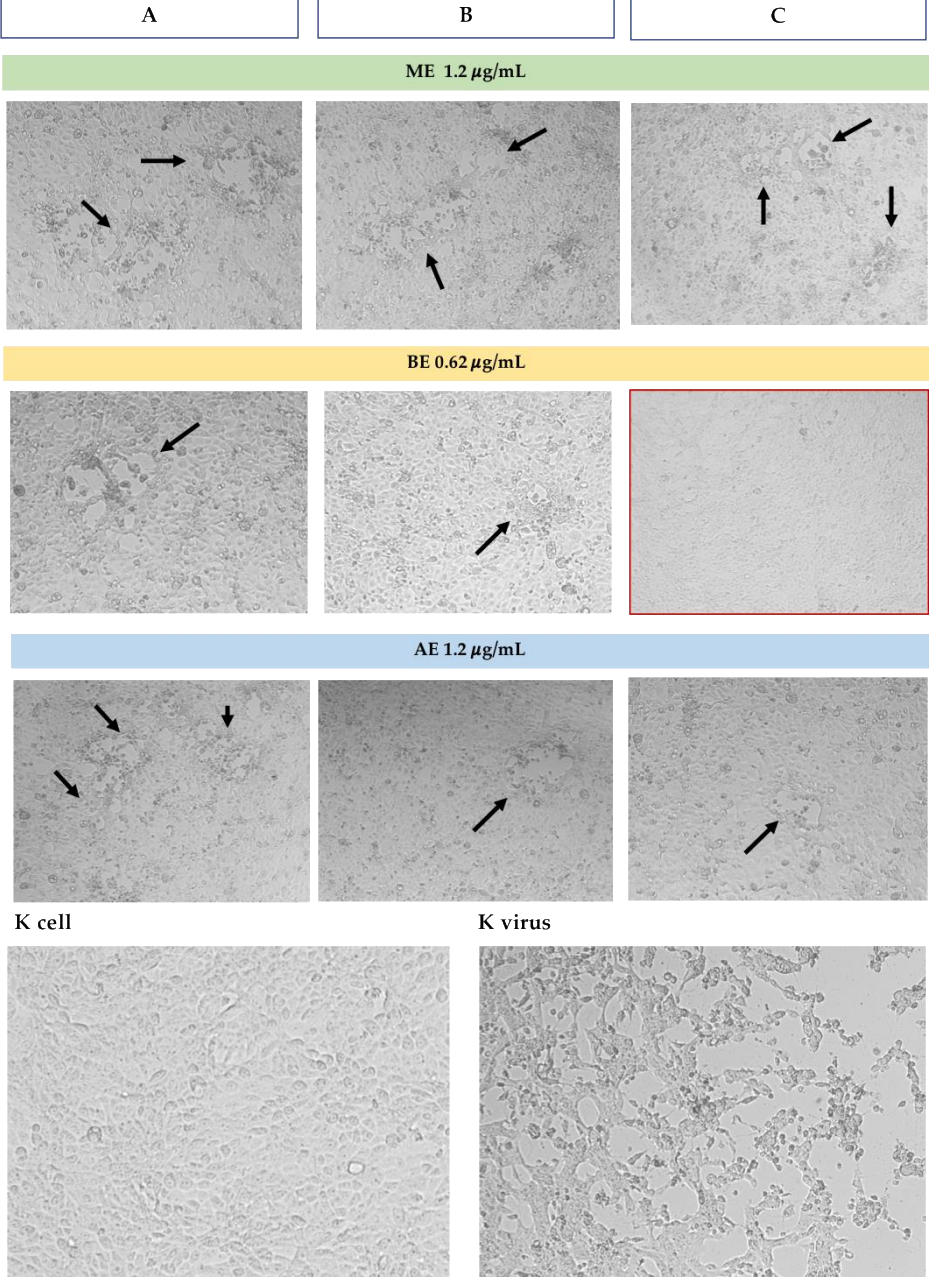
Microscopic observation of MDBK cell morphology (10×) in the three assays with the higher concentration of *H. bocconei* extracts. K cell: cell control (untreated), K virus: virus control (cells with CpAHV1). Black arrows indicate zones with cytopathic effect induced by CpAHV1. The red frame indicates the absence of cytopathic effect. Method (**A**): Cell culture pretreatment with *H. bocconei* extracts; (**B**): Treatment with *H. bocconei* extracts post virus infection; (**C**): Simultaneous viral infection and treatment of cell cultures with *H. bocconei* extracts.

Cell morphology observation was consistent with the increase in viability revealed by the MTT assay. No cytopathic effect induced by CpAHV1 was observed exclusively in the simultaneous treatment with BE (Figure 5).

#### 3.3.3. Antiviral Assays Against EqAHV1

Regarding the antiviral tests conducted against EqAHV1, a modest increase in RK-13 cell viability was observed only in the case of pretreatment with 0.62 μg/mL BE followed by viral infection (Method A). No *H. bocconei* extract caused significant changes in cell viability in the postinfection treatment (Method B).

**Figure 6:**
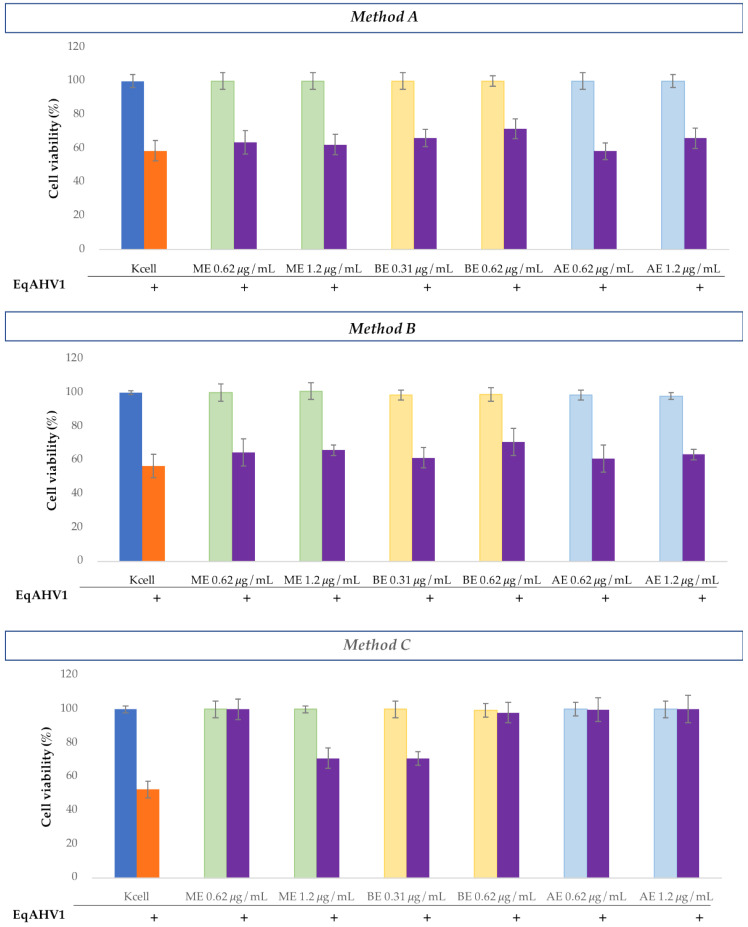
Cell viability (%) of RK-13 cells observed in the three experiments: **Method A**: Cell culture pretreatment with *H. bocconei* extracts; **Method B**: Treatment with *H. bocconei* extracts post virus infection; **Method C**: Simultaneous viral infection and treatment of cell cultures with *H. bocconei* extracts. Blue bar: RK-13 not treated; Orange bar: RK-13 infected with EqAHV1; Purple bar: cells treated with two different concentrations of each *H. bocconei* extract and infected with EqAHV1; Green bar: control of RK-13 cells treated with ME; Yellow bar: control of RK-13 cells treated with BE and Light blue: control of RK-13 cells treated with AE.

**Figure 7:**
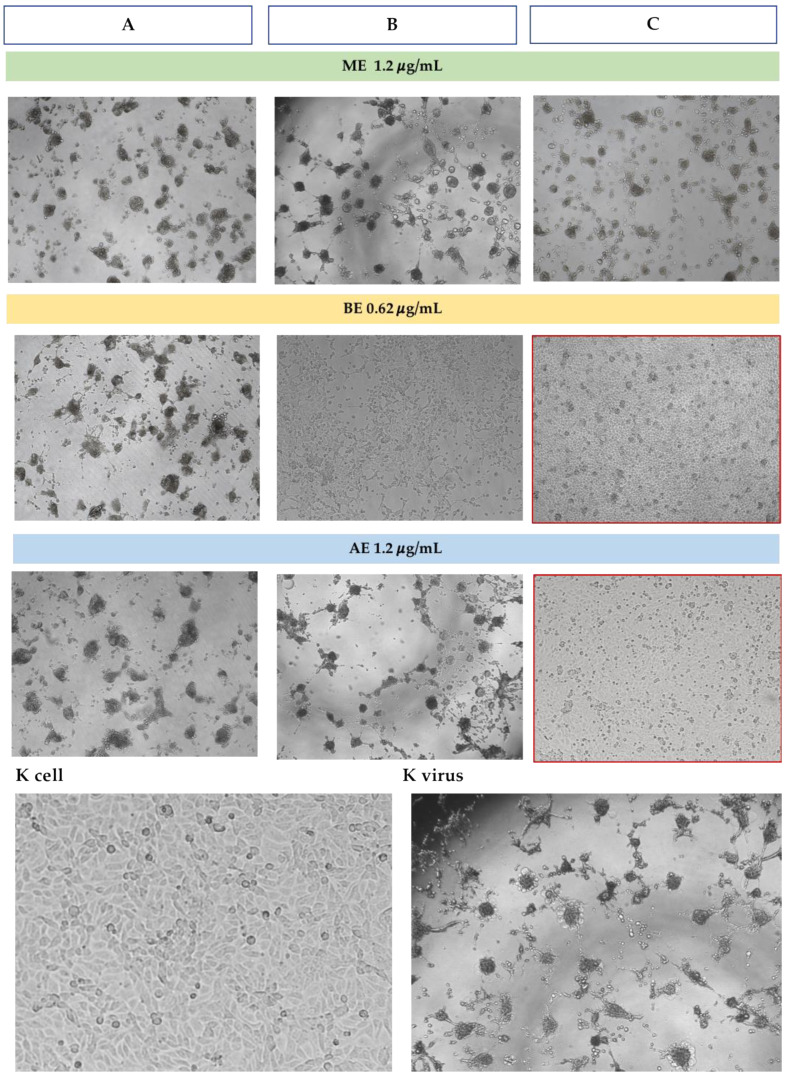
Microscopic observation of RK-13 cell morphology (10×) in the three assays with the higher concentration of *H. bocconei* extracts. K cell: cell control (untreated), K virus: virus control (cells with EqAHV1). The red frame indicates the absence of cytopathic effect. Method (**A**): Cell culture pretreatment with *H. bocconei* extracts; (**B**): Treatment with *H. bocconei* extracts post virus infection; (**C**): Simultaneous viral infection and treatment of cell cultures with *H. bocconei* extracts.

Regarding the Method C, all extracts induced a significant increase in cell viability, particularly with 0.62 μg/mL ME and BE, and with 0.62 and 1.2 μg/mL AE. The observation of cell morphology was consistent with the percentage increase in RK-13 cell viability revealed by the MTT test (Figure 6), as no cytopathic effect was observed in cells treated simultaneously with 0.62 μg/mL BE and 1.2 μg/mL AE (Figure 7).

## 4. Discussion

This study, for the first time, evaluated the antiviral properties of three different extracts obtained from the roots of *H. bocconei* against three animal herpesviruses: BoAHV1, CpAHV1, and EqAHV1, pathogens known to cause significant economic losses. Specifically, the results showed the anti-herpesvirus activity of the *n*-BuOH extract. *Helleborus* species have been reported for their biological activities. For example, the compound MCS-18 (macrocyclic carbon dioxide), isolated from *H. purpurascens*, has demonstrated an immunomodulatory effect in experimental autoimmune encephalomyelitis (EAE) [27]. Other species have been traditionally used for various disorders: *H. foetidus* for toothaches, *H. orientalis* for inducing abortion, *H. thibetanus* for cystitis, *H. odorus* for skin diseases, and *H. niger* for joint pain [23,44].

Regarding *H. bocconei,* an endemic plant of Sicily (Italy), previous studies have focused mainly on its chemical composition, cytotoxicity against rat glioma cells [38] and antibacterial activity [32].

The phytochemical data found in this study revealed that the *n*-BuOH extract from the roots of *H. bocconei* contains a mixture of ecdysteroids (ecdystenone and 20-hydroxyecdysone), helleborosides A and B, bufadienolides, and a combination of aliphatic and unsaturated components.

The results of the antiviral tests showed that the BE led to an increase in cell viability and a reduction in the cytopathic effect in cells infected with the three viruses and treated with *H. bocconei*, as well as in those treated with *H. bocconei* after viral infection, as well as in those treated with *H. bocconei* post viral infection.

Antivirals can interfere with one or more stages of the viral life multiplication cycle: cell attachment, cell penetration, viral uncoating, viral genome (DNA/RNA) replication, maturation and viral progeny release [15]. Currently, drugs used against herpesvirus inhibit viral replication or viral genome synthesis. To date, several phytochemicals have been reported for their antiviral activity against herpesviruses, including flavonoids, alkaloids, saponins, terpenes, quinones, lignans, polysaccharides, and tannins [45], substances abundant in *Helleborus* species [38]. Moreover, chemical analyses conducted on the roots of *Helleborus* have shown the richness in these species in polyphenolic compounds and thionines with antimicrobial and antiviral activities [46].

The absence of cytopathic effects in cells treated with *H. bocconei* BE (0.62 μg/mL) and infected at the same time individually with the three viruses suggests that the extract’s effect may act in the early stages of viral infection. These data may be due both to the activity of saponins (helleboroside A and helleboroside B), but also to polyphenols such as caffeic acid present in the roots of *H. bocconei* [47].

Herpesviruses utilize glycosaminoglycans (GAGs) as initial adhesion receptors during host cell infection. It has been shown that certain polyphenols can interact with viral glycoproteins responsible for binding to GAGs, thus preventing adhesion to the cell surface and subsequent recognition by entry receptors. This inhibitory effect appears to occur both during viral adhesion and fusion stages, as well as in the intercellular spread of the virus, as seen with herpes simplex virus type 1 (HSV-1), where viral glycoproteins mediate the process [45].

The caffeic acid inhibits the HSV-1 replication in vitro by interfering mainly with the early stages of multiplication in the infected cells prior to the completion of viral genome DNA replication [48].

Moreover, regarding the saponin presence, studies reported the antiviral activity of Saikosaponin B2, against a wide range of viruses, including the influenza A virus, hepatitis C virus, hepatitis B virus, measles virus, severe acute respiratory syndrome coronavirus 2 (SARS-CoV-2), respiratory syndrome virus and herpesvirus [49,50,51,52]. In particular, the Saikosaponin B2, was recently identified as potent antiviral drugs targeting the early entry of Feline herpesvirus-1 (FHV-1) by interacting with the viral surface functional protein gB. The gB protein is the most conserved glycoprotein in herpesviruses and is responsible for viral adsorption to the cell surface by binding to the acetylheparin sulfate receptor during viral infection of host cells [53,54].

The absence of cytopathic effects observed when cells were treated at the same time with *H. bocconei* BE and infected with each of the three herpesviruses (Method C) may suggest the possibility of conducting further in vivo studies to evaluate the potential use of the extract as a tool to complement the action of vaccines.

Another important result was the effect on the increase in cell viability in MDBK cells infected with CpAHV1 and subsequently treated with the *H. bocconei* BE (Method B). This suggests that the BE may also act on stages following the virus’s entry into the cell, such as viral genome replication, maturation, and release of new viral particles. These data may encourage future studies to further explore the role of bufadienolides, as similar compounds, such as bufalin, have been shown to inhibit viral replication [55].

## 5. Conclusions

This study is the first to demonstrate the antiviral activity of *H. bocconei* extracts, specifically highlighting the BE for its efficacy against animal herpesviruses BoAHV1, CpAHV1 and EqAHV1. Our results indicate that BE may prevent viral penetration and inhibit other stages of the viral life cycle.

Phytochemical analysis revealed that the BE comprises a complex mixture of bioactive compounds, including ecdysteroids, helleborosides, bufadienolides and various fatty acids, which may collectively contribute to its antiviral properties. These effects align with established mechanisms by which phytochemicals, such as polyphenols, interfere with viral attachment and replication by targeting glycoprotein interactions. Future research should focus on isolating the specific active constituents and elucidating their mechanisms of action, evaluating potential in vivo applications, and addressing any limitations, which could pave the way for novel therapeutic approaches in veterinary medicine.

## Figures and Tables

**Table 1:** Cytotoxic activities of MeOH (ME), *n*-BuOH (BE) and aqueous (AE) extracts on MDBK and RK-13 cells.

Cell Line	Concentration (µg/mL)	Mean Cell Survival Percentage (±SD)		
		ME	BE	AE
MDBK	0.31	101 (±0.1)	100 (±0.05)	100 (±1.03)
	0.62	100 (±0.13)	95 (±0.1)	100 (±0.9)
	1.25	97.68 (±0.12)	72.04 (±0.1)	99.63 (±0.14)
	2.5	59.79 (±0.15)	54.46 (±0.13)	77.12 (±0.12)
	25	38.14 (±0.05)	34.40 (±0.09)	44.62 (±0.05)
RK-13	0.31	100 (±0.06)	100 (±0.03)	100 (±0.02)
	0.62	100 (±0.01)	100 (±0.1)	100 (±0.06)
	1.25	100 (±0.04)	45.24 (±0.08)	100 (±0.08)
	2.5	98.23 (±0.09)	40 (±0.05)	100 (±0.1)
	25	60 (±0.08)	35.9 (±0.07)	50 (±0.1)

**Table 2:** The 50% cytotoxic concentration (CC_50_) values determined for each extract of *H. bocconei* assayed in MDBK and RK-13 cells.

Cell Line	Extract	CC50 (µg/mL)
MDBK	ME	12.67
	BE	7.32
	AE	21.27
RK-13	ME	>25
	BE	1.19
	AE	25

## Data Availability

Data are contained within the article.

## Abbreviations

The following abbreviations are used in this manuscript:

ME	MeOH extract
BE	*n*-BuOH extract
AE	Aqueous extract
